# Dielectric Imaging of Fixed HeLa Cells by In-Liquid Scanning Dielectric Force Volume Microscopy

**DOI:** 10.3390/nano11061402

**Published:** 2021-05-25

**Authors:** Martí Checa, Ruben Millan-Solsona, Adrianna Glinkowska Mares, Silvia Pujals, Gabriel Gomila

**Affiliations:** 1 Nanoscale Bioelectric Characterization, Institut de Bioenginyeria de Catalunya (IBEC), The Barcelona Institute of Science and Technology (BIST), c/Baldiri I Reixac 11-15, 08028 Barcelona, Spain; rmillan@ibecbarcelona.eu; 2 Departament d’Enginyeria Electrònica i Biomèdica, Universitat de Barcelona, c/Martí i Franquès 1, 08028 Barcelona, Spain; spujals@ibecbarcelona.eu; 3 Nanoscopy for Nanomedicine, Institut de Bioenginyeria de Catalunya (IBEC), The Barcelona Institute of Science and Technology (BIST), c/Baldiri I Reixac 11-15, 08028 Barcelona, Spain; aglinkowska@ibecbarcelona.eu

**Keywords:** scanning probe microscopy (SPM), electrostatic force microscopy (EFM), scanning dielectric microscopy (SDM), atomic force microscopy (AFM), dielectric properties, functional microscopy

## Abstract

Mapping the dielectric properties of cells with nanoscale spatial resolution can be an important tool in nanomedicine and nanotoxicity analysis, which can complement structural and mechanical nanoscale measurements. Recently we have shown that dielectric constant maps can be obtained on dried fixed cells in air environment by means of scanning dielectric force volume microscopy. Here, we demonstrate that such measurements can also be performed in the much more challenging case of fixed cells in liquid environment. Performing the measurements in liquid media contributes to preserve better the structure of the fixed cells, while also enabling accessing the local dielectric properties under fully hydrated conditions. The results shown in this work pave the way to address the nanoscale dielectric imaging of living cells, for which still further developments are required, as discussed here.

## 1. Introduction

The dielectric properties of cells have been the subject of intense research over the years due to its relevance in many fundamental and applied biological problems. At the whole cell level, changes in the dielectric properties of cells have been studied as possible cancer markers [1] or cell apoptosis indicators [2,3], showing the relevance of such characterization in the medical field. At the subcellular level, changes in the dielectric properties of the cell membrane have been shown to play a crucial role in membrane charge storage and charge separation [4], crucial for many intra and inter cellular processes involving electrostatic interactions.

The measurement of the dielectric properties at the whole cell level can be done through electrokinetic techniques, such as dielectrophoresis [5], electrorotation [6] or impedance cytometry [7]. Nevertheless, such techniques offer a limited description of the dielectric properties of cells at the local level and, hence, provide averaged values of the spatial variations that take place along the cell itself. Optical techniques involving polarization-dependent [8], voltage-dependent [9] or ionic-dependent [10] fluorescence labels enable obtaining local dielectric information on cells, overcoming the spatial resolution limitation of the electrokinetic measurements. However, these images are difficult to analyze in terms of physical properties such as dielectric constant or conductivity since they generally lack models for its quantification.

Impedance-based measurements can provide also local dielectric information by reducing the measuring electrode size via patterned microelectrode array planar devices (MEAs) [11,12], in liquid working transistors [13] or micro positioned micro and nanoelectrodes, like in scanning ion conductance or scanning electrochemical microscopy [14,15,16] or in scanning dielectric microscopy in current sensing mode [17,18]. However, achieving quantitative dielectric sub-micrometric spatial resolution still remains challenging, which results in a lack of complete studies of the subcellular dielectric mapping, especially for small and delicate cell structures, like axons in neurons or other cell appendages [19]. The use of microwaves (GHz) in the near field probed by small conductive antenna, as in scanning microwave microscopy (SMM), have also been explored for dielectric imaging of cells in liquid environment. Nevertheless, such studies are sometimes restricted to the use of non-polar solvents [20] and/or lack of a quantitative analysis [21]. 

Electric force-based scanning probe microscopy (SPM techniques, such as scanning dielectric microscopy (SDM) in force detection mode [22], offer an alternative route to map the dielectric properties of cells, as it has been demonstrated already for cells in dry conditions [23,24,25,26,27]. The extension of this approach to cells in liquid conditions; however, is not immediate since SDM in liquids triggers a complex frequency and voltage dependent response in the system [28], involving phenomena such as ionic migration, surface stress or chemical reactions (especially when low frequency and high voltages are used in their operation). Ionic charge screening due to the formation of electrostatic double layers (EDL) is the main issue preventing SDM operation in polar solutions. In non-polar solvents, SDM is easier to apply [29], since ionic screening does not occur. However, these solvents are less relevant for biological applications. To shortcut these issues, smart novel methods where developed, like electrochemical force microscopy (EcFM) [30,31], open-loop Kelvin Probe Force Microscopy (OL-KPFM) [32,33] or in-liquid SDM in force detection mode (in-liquid SDM) [34,35,36,37,38,39]. These techniques overcome ionic charge screening by using different strategies, such as by performing the measurement of the transient behavior after an excitation, by positioning the tip at a very close distance from the sample (shorter than the characteristic Debye screening length, λ_D_) or by applying a high frequency electric potential, higher than the dielectric relaxation frequency of the electrolyte. Nevertheless, to the best of our knowledge, such modes have not yet been applied to the challenging case of the nanoscale dielectric imaging of cells in liquid environment.

In this paper we will show how in-liquid SDM, which has already been successfully applied to study the capacitive properties of self-assembled monolayers (SAMs) [36], lipid bilayer patches [38,39], liposomes [40] and functional organic thin film transistors [37] in electrolyte solutions, can be applied to map the dielectric properties of fixed cells in electrolyte solutions. The measurements are done through the implementation of a fast force-volumetric mode (in-liquid SDFVM [27,37]), which avoids the application of lateral forces (extremely important for weekly adhered, and soft samples like cells), and enables an accurate mapping of the local dielectric properties. By using this approach, local equivalent dielectric constant maps of the fixed cells in the electrolyte solutions have been derived. It is shown that the maps provide information on both the local water content of the cell and its biochemical composition.

## 2. Materials and Methods

### 2.1. Sample Preparation

HeLa cells from a lab cell line seeded on top of gold-coated silicon substrates were used in the present study. The protocol to prepare the sample was optimized from the different options shown in [41], and it is similar to the one used in [26], except that the imaging is done here under an electrolyte solution. In a nutshell, the cells (passage 10) were seeded at 50k cells/mL onto the gold-coated silicon substrates (Arrandee metal GmbH, Westfalen, Germany) placed in a petri dish with Dulbecco’s modified Eagle medium (DMEM, as received with L-Glutamine, 4.5 g/L D-glucose and pyruvate, Gibco, Fisher Scientific, SL, Madrid, Spain) supplemented with Fetal Bovine Serum (FBS) 10% (Gibco, Fisher Scientific, SL, Madrid, Spain) and penicillin/streptomycin 1% (Biowest SAS, Nuaillé, France). After 24 h of incubation at 5% CO_2_ and 37 ℃, the cells were observed and seemed completely attached and spread on the gold substrate. Then, cells were fixed. The medium was washed with PBS, and the cells were incubated with 2.5% glutaraldehyde in PBS for 10 min. After fixation, the cells were washed consecutively with different solutions: 75%, 50% and 25% PBS, ultrapure MilliQ water, and 10 mM 3-(N-morpholino)propanesulfonic acid (MOPS ) buffer. Prior to the Atomic Force Microscopy (AFM) experiments, the cells and substrate were moved from the petri dish to the AFM sample holder and a drop of 10 mM MOPS buffer was placed onto the sample to perform the experiments in fully hydrated conditions.

### 2.2. In-Liquid SDFVM

**Figure 1:**
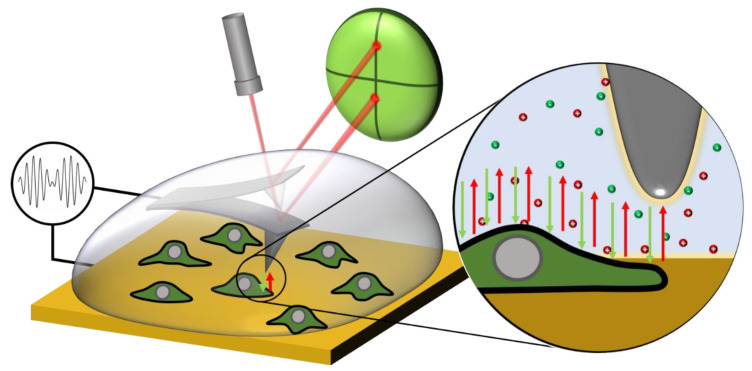
Sketch of the experimental setup for in-liquid SDFVM for the dielectric imaging of fixed HeLa cells in electrolyte solutions.

In-liquid SDFVM measurements were done following the methods of in-liquid SDM [34,35,36,38,39] extended to work in the force-volume mode [27] in liquid media [37]. Figure 1 shows schematically the experimental setup used for in-liquid SDFVM applied to the imaging of cells. An amplitude modulated high frequency voltage of the form $V\left(t\right)=\frac{V_{\mathrm{AC}}}{2}\left(1+\cos\left(\omega_{\mathrm{mod}}t\right)\right)\cos\left(\omega_{\mathrm{el}}t\right)$, with f_mod_ = 5 kHz and f_el_ = 5 MHz, was applied between a conductive tip (NSC19/Cr–Au MikroMasch, Tallinn, Estonia, k∼0.5–1.5 N m^−1^, f_res_∼68 kHz) and the metallic substrate. The value of k was determined by means of the thermal noise method. The relatively large uncertainties in the determination of k (up to a 20%) can affect the extracted geometric parameters of the tip but they do not affect remarkably the extracted homogeneous dielectric constants. The choice of f_el_ was done prior to the experiments by selecting a frequency above the critical relaxation frequency of the solution [42], and below 500 MHz to avoid high-frequency impedance mismatches produced in the conventional non-shielded circuitry. The applied voltage induces the static bending of the probe and oscillations at different harmonics of the modulation frequency (for further details see, for instance, [36]). Force-distance curves are acquired at each point of the sample of interest, while both the static deflection of the cantilever D(X,Y;Z) and the amplitude of the first harmonic of the modulated electrical oscillation of the tip A_ωmod_(X,Y;Z) are acquired. 

The deflection of the cantilever is used to determine the contact point with the sample and hence determine the sample’s topography, while the oscillation amplitude is used to measure the derivative of the capacitance of the system, dC/dz, referred to as the capacitance gradient, through the following relationship $\frac{\mathrm{dC}}{\mathrm{dz}}=\frac{8k\left(A_{\omega \mathrm{mod}}\right)}{V_{\mathrm{AC}}^{2}\mathrm{mG}}$, where k is the cantilever equivalent spring constant, m the photodiode sensitivity and G the gain of the Lockin Amplifier (for more details see Ref. [36]).

The operation of in-liquid SDM in the fast force-volumetric scan mode prevents the application of lateral forces to the sample, crucial in the case of weekly adhered samples like cells. Nevertheless, for the case of extremely soft samples (like living cells), this mode can add an extra complication to the data processing, since the determination of the tip-sample contact point is not straightforward, thus the unperturbed sample’s topography can be difficult to reconstruct (see [43,44] and the Discussion section). This is not an issue in the case of fixed cell samples, since the fixation induces cell hardening, which makes easy to identify the tip-sample contact point and hence the unperturbed topography of the sample (while it preserves reasonably well the cell structure and composition). 

A custom-made software written in Matlab (Mathworks) enables building up the so-called Data Cubes from the ensemble of acquired electrical deflection and oscillation curves, from where, all the different classical electrostatic force microscopy (EFM) images can be retrieved (e.g., lift mode or constant plane images) [27].

### 2.3. Dielectric Constant Mapping

**Figure 2:**
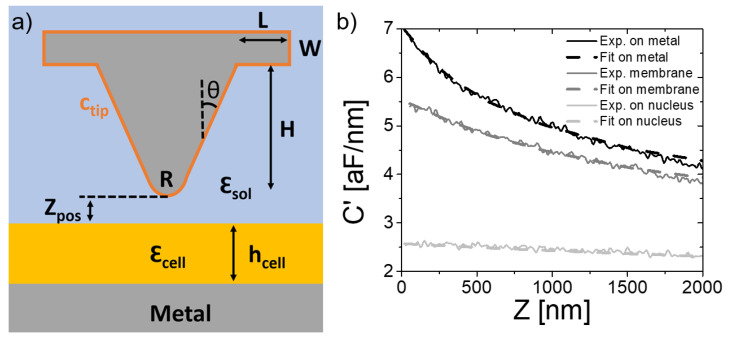
(**a**) Sketch of the thick-film model used for the quantification of the in-liquid SDFVM measurements in cells in electrolyte solutions, with the different parameters highlighted. (**b**) (continuous lines) Examples of experimental capacitance gradient–distance curves acquired on top of the metal (black continuous line), cell membrane (grey) and cell nucleus (light grey), corresponding, respectively, to pixels (21, 116), (77, 95) and (66, 53) in Figure 3. The grey dashed lines correspond to the theoretical fits performed using the model in (a), giving dielectric constants ε_cell_ = 36 ± 1 and ε_cell_ = 77 ± 5. The black dashed line corresponds to the fit of a tip-metal substrate theoretical model, giving a tip radius, R = 5 nm, substrate capacitance, c_subs_ = 0.425 µF/cm^2^, and capacitance gradient offset, C’_offset_ = 1.1 aF/nm, respectively. Modelling parameters—θ = 20 deg and c_tip_ = 2.7 µF/cm^2^.

We determined the local equivalent homogeneous dielectric constant of the cells in electrolyte media by using a local thick-film dielectric model [45]. This model is expected to work better in liquid media than in air due to the broadening of the field lines in the liquid environment. The model consists of a tip in front of a laterally infinite homogeneous thick dielectric film with thickness h_cell_, equal to the local thickness of the cell, and dielectric constant, ε_cell_, to be determined (see Figure 2a). The environment is assumed to have dielectric constant ε_sol_. Ionic conductivity effects are not included since the frequency of the applied voltage (f_el_ = 5 MHz) is larger than the dielectric relaxation frequency of the electrolyte solution (f_RC_ = 0.5 MHz), that can be calculated via conductivity (σ_sol_) measurements as: $f_{\mathrm{RC}}=\frac{\sigma_{\mathrm{sol}}}{2{\pi \varepsilon }_{0}\varepsilon_{\mathrm{sol}}}$. The tip is modeled as in previous works [22] by a cone of height H and half angle θ ended with a tangent sphere of radius R and caped with a disc of thickness W and radius L+H·tan(θ). Interfacial capacitances, c_tip_ = 2.7 µF/cm^2^ and c_subs_ are added to the tip and bare substrate parts to model interfacial effects in electrolytes, as described elsewhere [36]. The microscopic tip parameters are left to their nominal values H = 12.5 µm, W = 3 µm, L = 3 µm, θ = 20 °, while the radius and tip interfacial capacitance (and the capacitance gradient offset) are determined from capacitance gradient approach curves acquired on the bare substrate, as described earlier [22]. With this set of parameters, capacitance gradient–distance approach curves are calculated for different values of the dielectric constant of the film and different thicknesses and are fitted to the experimental capacitance–gradient approach curves acquired at each position in the cell, with the film dielectric constant as single fitting parameter. Examples of fitted and experimental curves for three different positions in a sample (substrate, cell membrane, cell nucleus) are shown in Figure 2b. The theoretical capacitance gradient values are obtained from the calculated electric force acting on the tip, which in turn is obtained by integration of the Maxwell-stress tensor on the tip surface. The calculations were done by using the electric currents module in COMSOL Multiphysics 5.4 (COMSOL Inc., Burlington, MA USA), as explained elsewhere [36].

## 3. Results

**Figure 3:**
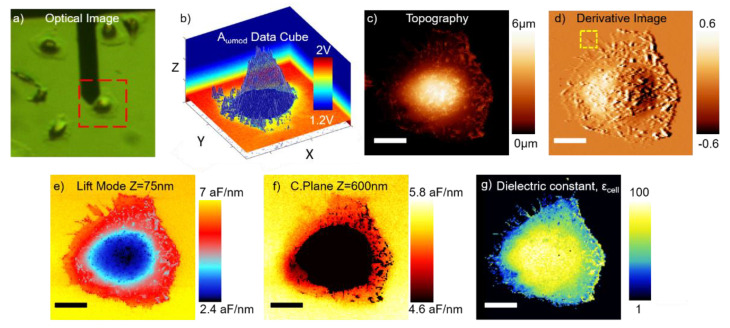
(**a**) Optical microscopy image of the fixed HeLa cells in MOPS buffer with the SDM probe positioned on one of them. The red-dashed square highlights the zone imaged with the in-liquid SDM. (**b**) A_ωmod_ Data Cube built up from the ensemble of in-liquid SDM force-distance curves. (**c**) Topography image. (**d**) Derivative of the topography image (highlights the topographic edges). (**e**) Lift Mode SDM image at a lift distance of 75 nm. (**f**) Constant Plane SDM image at a distance 600 nm from the substrate. (**g**) Dielectric constant image of the cell. XY Scalebar in the images is 12 µm. Experimental parameters—128 × 128 pixels, V_AC_ = 1 V, f_mod_ = 5 kHz, f_el_ = 5 MHz, k = 1.2 N/m. Modelling parameters—R = 5 nm, θ = 20 deg, c_tip_ = 2.7 µF/cm^2^, C’_offset_ = 1.1 aF/nm and c_subs_ = 0.425 µF/cm^2^.

Figure 3 shows the in-liquid SDM imaging of a fixed HeLa cell in MOPS buffer. Figure 3a is an optical image of the tip positioned on top of the cell, with the red dashed rectangle highlighting the scanned area. Figure 3b shows the oscillation amplitude Data Cube built up from the ensemble of electrical oscillation force distance curves acquired at each pixel of the image (A_ω_(X,Y;Z)). Figure 3c presents the topography of the cell obtained from the normal deflection Data Cube (not shown). The cell presents a very heterogeneous topography, with heights ranging from around 400 nm in the regions where the membrane is extended, to around 6 µm at the taller parts where the nucleus stands. These dimensions are close to the one displayed by non-fixed cells, indicating that the cell is fully hydrated (for non-rehydrated fixed cells the water evaporation induces a cell shrinkage with heights in the range from 200 nm up to 1 µm, see [26]). Figure 3d shows the derivative image (also known as error image), which better highlights the edges of the cell structure, which are sometimes difficult to see under the same color-scale in a topography image with a wide range of height variation.

Figure 3e shows the lift mode image at a tip-sample distance of 75 nm, reconstructed from the oscillation amplitude Data Cube in Figure 3b, following the exact same procedure as in [27]. A huge electric contrast is found between the different zones of the cell in the lift mode image. However, the contrast comes mainly from topographic crosstalk effects, as the tip-substrate distance varies a lot during the scan. Therefore, this image is not fully related to the local dielectric properties of the cell, as we discussed in detail elsewhere [46]. We show it in here for the sake of completeness, as it is the most common imaging mode used in conventional EFM imaging. Figure 3f shows a constant plane image at a tip-substrate distance of 600 nm, also reconstructed from the oscillation amplitude Data Cube in Figure 3b. The black region does not contain data since it is located at a height above the imaging height. This image is free from the topographic crosstalk artifact commented previously, as the tip-substrate distance is maintained constant along the electrical scan. The images show a negative contrast (i.e., the electric force on top of the cell is smaller than on top of the substrate at the same height). This fact implies that the equivalent homogeneous dielectric constant of the cell, ε_cell_, is lower than the dielectric constant of the electrolyte solution, ε_sol_. Therefore, despite the cell being basically filled with electrolyte solution, still the dielectric images can sense the dielectric contribution of the cell components. Nevertheless, dC/dz constant plane images are not yet dielectric constant maps. To derive them, one needs to consider the local geometrical features of the cell, such as the thickness, shape, and internal structure.

To generate the dielectric constant map, we used here a local thick-dielectric model with calibrated tip geometrical parameters (see the limitations of the model in the discussion section, and Materials and Methods). Figure 3g shows the dielectric constant map obtained for the cell in the present case. The dielectric constant map must be understood as representing the local equivalent homogeneous dielectric constant of a small cell volume located below the tip. The local dielectric constant values, ε_cell_, span a wide range of values from ≈20 in the cell extended parts to ≈80 in the central part. As advanced before, most of these values are smaller than the dielectric constant of the solution ε_sol_ = 80, from where the negative contrast in the constant plane dielectric images. In the Discussion section, we explain the origin of the wide range of values obtained and the information that can be obtained from them concerning the cell dielectric properties.

**Figure 4:**
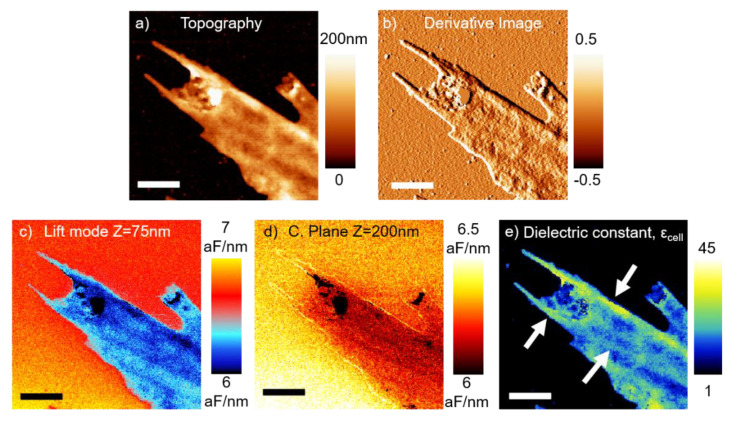
(**a**) Topography image of the zoomed-in region highlighted with the yellow dashed rectangle in Figure 3d. (**b**) Derivative image. (**c**) Lift mode SDM image at a lift distance of 75 nm. (**d**) Constant Plane SDM image at a distance 200 nm. (**e**) Dielectric constant image of the cell. Note that the color scale range is different from that in Figure 3g. XY scalebar is 1 µm. Experimental parameters—same as in Figure 3, except the pixel number, which in this case is 256 × 256 pixels. Modelling parameters—same as in Figure 3.

Figure 4 shows a higher resolution zoomed-in image acquired on a cell membrane extension of the cell in Figure 3. Figure 4a displays the topography of the zone highlighted with a yellow rectangle in Figure 3d. Here, we can see with greater detail one of the cytoplasmatic projections of the cell (presumably lamellipodia or filopodia, crucial for cell migration). Figure 4b is the consequent derivative image, where the edges of the structure are clearly highlighted. Already in the topographic image, some subsurface fibers (probably cytoskeletal actin filaments) seem to drive the structure of the cell elongation. Figure 4c,d shows the lift mode and constant plane SDM images of that zone, respectively. Again, the appearance of some contrast in constant plane images ensures for a distinct dielectric polarizability of the cell with respect to the electrolyte. Figure 4e displays the equivalent homogeneous dielectric constant map, where the zone with cytoskeleton fibers (some of which are marked with white arrows) appears to have a higher dielectric signal than the regions between fibers.

## 4. Discussion

We derived effective local dielectric constant maps of fixed HeLa cells in an electrolyte solution, by means of the cutting-edge advances of in-liquid SDFVM, based here, on a simple thick-film model for their quantification. The dielectric constants obtained with this approach represent the equivalent homogeneous dielectric constant of a small volume located below the tip at each sample position. Therefore, a combination of both, the local dielectric constant of the cell biomolecular components (i.e., lipids, proteins, and nucleic acids) and the water content of the cell, is simultaneously mapped. The equivalent homogeneous dielectric constant obtained is used to identify the presence of internal heterogenous dielectric properties.

**Figure 5:**
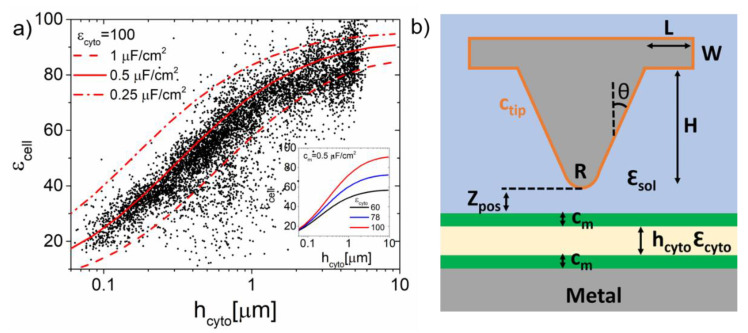
(**a**) (Symbols) Equivalent homogeneous dielectric constant values of the cell in Figure 3g as a function of the height of the cytoplasm (which is assumed to be the height of the cell minus twice the thickness of the membrane). (Lines) Equivalent homogeneous dielectric constant as a function of the cytoplasm height predicted for the core-shell tip-thick dielectric model in (**b**) for three different values of the specific capacitance of the cell membrane (c_m_ = 0.25, 0.5 and 1 μF/cm^2^) and a value of the dielectric constant of the cytoplasmatic region (ε_cyto_ = 100). Inset: Equivalent homogeneous dielectric constant as a function of the height of the cytoplasm predicted by the model in (**b**) for a membrane specific capacitance c_m_ = 0.5 μF/cm^2^ and three different values of the dielectric constant of the cytoplasm (ε_cyto_ = 60, 78, 100). (**b**) Tip-core-shell thick dielectric model, with the relevant parameters highlighted. Parameters of the tip used in the calculations—same as in Figure 3.

Figure 5a (symbols) shows a plot of the ε_cell_ values as a function of the height of the cell. There is an obvious correlation between the dielectric constant values and the height (thickness) of the cell, which automatically implies that some internal dielectric heterogeneity should be present. For a cell, the main internal dielectric heterogeneity is given by the presence of the cell membrane, the cytoplasmic region (containing the organelles and other sub-cellular structures) and water. Due to the high dielectric constant of water, the contribution of the cytoplasmic structures in the volume probed by the tip, in a first approximation, can be taken as a small correction to the dielectric response of water.

To support this interpretation, we considered a “core-shell” thick dielectric model, with two thin layers of specific capacitance c_m_ = ε_0_·ε_m_/h_m_, where h_m_ and ε_m_ are the thickness and dielectric constant of the layer, respectively, which represent the cell membrane and an internal thick layer of thickness h_cyto_ and dielectric constant ε_cyto_ representing the cytoplasmatic region (see Figure 5b). Figure 5a (continuous lines) shows the equivalent dielectric constant predicted for this model as a function of the thickness of the cytoplasmatic region, for ε_cyto_ = 100 and three different values of the specific capacitance of the cell membrane c_m_ = 0.25, 0.5, 1 μF/cm^2^. The theoretically calculated values (lines) follow nicely the trend of the experimental values. The values obtained for both parameters are within the range of values reported in the literature for eukaryotic cells, what is quite remarkable. This result indicates that the dielectric constant maps can provide information on both the dielectric properties of the cell content and of the cell membrane. We note that the cell content is expected to be composed of electrolyte solution, but due to the crowded nature of the cell cytoplasm it may happen that its actual dielectric constant differs from that supposed for water and would resemble more to the dielectric constant of a biological solution with proteins, lipids, DNA, etc., at the frequency of the measurements. The effect of the variations of ε_cyto_ predicted by the model is shown in the inset of Figure 5a. 

The previous analysis explains why, in Figure 3g, we observe a higher dielectric response on the tall center of the cell and smaller ones towards the cell edges, where the cell membrane is more stretched/extended. We assume that this is primarily a direct consequence of the higher relative electrolyte volume (with respect to cell biomolecule volume) found in the center of the cell with respect to the edges. Similarly, in Figure 4e, we observe an overall lower dielectric response, because we are on top of the extended membrane part of the cell which is thin and implies less solution volume beneath the tip than in the nuclear zone, which is the main source of polarization. However, the better spatial resolution of this image allows us to distinguish different polarization zones. Remarkably, a higher dielectric response is found in the regions along the cytoskeleton fibers (marked with white arrows), that are clearly distinguished already in the topography image. We attribute this higher polarizability regions to a mixed solution-composition response. The regions along the cytoskeleton fibers, display a higher topography, which means more relative electrolyte volume beneath the cell at that points. In addition, cytoskeleton fibers should have a higher proteinic composition with respect to pure membrane regions, where lipids are expected to be predominant. Both reasons point towards a higher local polarization of such structural cell parts (as found experimentally). Additional theoretical models to disentangle each individual contribution would be required, which we consider to be out of the scope of this work.

Contrary to what happens in the case of the dielectric mapping of fixed cells in dry conditions [26], where the absence of water (highly polarizable) allows to directly correlate the dielectric maps with biochemical composition maps using the dielectric fingerprint of each cell biomolecular component separately, in the case of measuring in liquid environment, such direct correlation is not as straightforward, and the analysis becomes more complex. Therefore, the effective dielectric maps are now easier to analyze in terms of polarization differences with respect to the dielectric response of the electrolyte solution itself, assumed to be ε_solution_ ≈ 80. Namely, if the dielectric response of a region is smaller than the one of the solutions, that means that the detection of some low polarizable biocomponent of the cell is affecting the local polarizability. Likewise, if the dielectric response of a region is higher than the one of the solutions, it implies some higher polarizability of the region. The main sources of change in the dielectric response can be identified as either different local dielectric response of the different biochemical compounds or as different amount of solution volume in the region the tip is sensitive to. Other sources of changes in the dielectric response could also be assigned to local conductivity changes that can arise mixed resistive/capacitive response or different local adsorbates present in the cell surface as stated above. For the measurements reported here on fixed cells, for which the internal and external solution should have a similar ionic composition, we expect conductivity effects to be neglectable (if any), since the measurements are made at frequencies larger than the dielectric relaxation frequency of the electrolyte.

In addition to what is said above, we must consider at least three potential sources of error in the dielectric constant determination in the present work: The non-realistic geometrical model used (especially at the non-planar parts of the cell), the small conductivity effects if the intracellular media remains with a higher molarity than the extracellular solution (which would turn into an overestimation of the dielectric constant) and the error in the contact point determination (which would induce small changes in the h_cell_ considered in the model, inducing an overestimation or underestimation of the dielectric constant value depending on whether the true topography is higher or lower than the measured one).

Determining the exact lateral resolution of the technique is not straightforward, as it depends on many different factors, including pixel size, tip radius and cone angle, local geometry and thickness, and the dielectric response of the environment. For the case of large-scale images (like the one in Figure 3), the resolution is given by the pixel size (≈450 nm in this case). However, this is not the limit of the technique. For the case of the image in Figure 4 (where the pixel size is drastically reduced to 23 nm), we approach such limit with the goal of probing the smaller cell features (i.e., cytoskeleton fibers), which are in the nanometric range. A full theoretical analysis of the electric force contributions to the total electric force in in-liquid SDM, similar to the one we did in the past for SDM in air [47], would be required to accurately determine the resolution limit of the technique and is still pending. As a rule of thumb, for low polarizable environments, like air, a good estimation of the spatial resolution is the order of magnitude of the tip radius. However, for high polarizable environments, like the case of water-based solutions, the resolution is normally coarser, as the relative signal collected through the cone and cantilever of the tip increases as the dielectric constant of the media is raised. For the conditions and parameters of the present study, we have estimated by means of the numerical calculations that the cone and tip apex parts of the probe account, respectively, for around 45% and 55% of the electric force variations observed in the images, resulting in a lowering of the spatial resolution. That said, we have recently shown in [36] that a spatial resolution down to 50 nm can be achieved in planar samples with in-liquid SDM. 

The potential of SPM mechanical and dielectric characterization to obtain subsurface information has been widely proven [48,49,50,51], and in the case of cells could be specifically relevant to gather knowledge of internal structure such as smaller cell organelles or internal cell biological processes [51]. The force-volumetric approach used in this work could enable obtaining both local mechanical and dielectric information on the cell simultaneously. To this end, one would need to analyze the normal deflection curves in addition to the oscillation amplitude curves, acquired simultaneously. The normal deflection curves are used here to obtain the topographic image by setting a given set-point, but mechanical information could also be obtained by analyzing further the contact part of the normal deflection curves. Since fixation [52] induces hardening of the cells, as compared with living cells, there is not real interest in the mechanical analysis in this case. In any case, like in the quantification of the dielectric measurements, several factors can affect the mechanical quantification [53]. Performing such a detailed and complex analysis lies outside the scope of the present work. 

The extension of the proposed approach to the case of living cells still needs further investigation. Living cells are delicate samples and need to be maintained in specific electrolyte media to keep its structure and function, and with controlled temperature (37 ℃) and CO_2_ (5%). Cell media for living cells experiments are normally rich in ions to avoid an osmotic shock and also in nutrients necessary to maintain cell functions. However, the presence of large amounts of mobile charges in the electrolyte may hinder the operation of in-liquid SDM, limited by its operation at frequencies larger than the electrolyte relaxation frequency, which escalates quickly with solute molarity. Thus, a balance must be achieved for the realization of the experiments between preserving cell structure and being able to measure its dielectric properties with in-liquid SDM. Typically, using the experimental setup we implemented for this work, allows to reach the 10–100 MHz frequency range of applied electric voltage, and considering other similar implementations one can even reach the GHz range if a refinement of the electric shielding circuitry is carried out [54]. This experimental limitation sets a limit for the molarity of the solution to be used, which for our case is in the order of 10–20 mM range (this number depends also on the chemical composition of the solute), but could possibly be extended to 100’s mM if the GHz frequencies are used. 

The extension of the proposed method to the dielectric imaging of living cells comes with other additional adversities, such as the complication of the determination of the “true-topography” of the cell or the presence of active cell movements during the scan times. If such struggles are overcome, we envision in-liquid SDM to be an excellent tool to map both static and slow-dynamic biological processes happening at the cellular and subcellular scale, challenging to access by means of any other state-of-the-art electrochemical, optical or impedance-based methods, which show overwhelmingly large parasitic capacitive contributions when implemented with sub-micrometric electrodes.

## 5. Conclusions

We applied the recent developments of in-liquid scanning dielectric microscopy in force-volume mode to the challenging case of imaging the dielectric properties of fixed eukaryotic cells in electrolyte solutions. We derived local equivalent homogeneous dielectric constant maps of the cells with nanometric spatial resolution and showed that they provide information on both the water content of the cell and the dielectric properties of the cell membrane and cytoplasmatic content. The results presented here pave the way for the more interesting case of dielectric imaging of living cells at the nanoscale, where dielectric changes during biological functional processes could be potentially studied.

## Data Availability

The data presented in this study are available on request from the corresponding author.
